# MOVI-daFIT! Intervention: Rationale and design of a cluster randomized controlled trial testing the effects on improving adiposity, cognition, and subclinical atherosclerosis by increasing cardiorespiratory fitness in children

**DOI:** 10.1097/MD.0000000000014737

**Published:** 2019-03-01

**Authors:** Vicente Martínez-Vizcaíno, Celia Álvarez-Bueno, Iván Cavero-Redondo, Diana P. Pozuelo-Carrascosa, Miriam Garrido-Miguel, Jose Alberto Martínez-Hortelano, Vanesa Martínez-Madrid, Enrique Prada de Medio, Mairena Sánchez-López

**Affiliations:** a Universidad de Castilla-La Mancha, Health and Social Research Center, Cuenca, Spain; b Universidad Autónoma de Chile, Facultad de Ciencias de la Salud, Talca, Chile; c Servicio de análisis clínicos, Hospital Virgen de la Luz, Cuenca, España; d Universidad de Castilla-La Mancha, School of Education, Ciudad Real, Spain.

**Keywords:** academic performance, after-school program, cardiovascular risk, cognition, physical activity, school children

## Abstract

**Introduction:**

High-intensity interval training (HIIT) programs have demonstrated positive effects on cardiorespiratory fitness and cardiometabolic parameters, but their impact on other health parameters (such as body mass and fat) and cognition remains unclear. This paper presents the rationale and methods of a HIIT after-school physical activity (PA) intervention (MOVI-daFIT!) on reducing fat mass and cardiovascular risk, and improving physical fitness, executive function, and academic achievement among children aged 9 to 11 years old.

**Methods:**

A cluster randomized controlled trial (RCT), including 10 schools from Cuenca province, Spain, was designed. Schools were randomly assigned to the MOVI-daFIT! intervention and to the control group. Children were evaluated at the beginning (September 2017) and at the end (June 2018) of the school year. Children in the intervention group were involved in 60-minute after-school sessions 4 days per week developed in the school setting. Each session consisted of 15 minutes of set-up and warm-up games, 28 minutes of games using the HIIT protocol, and 10 minutes of cool down. In addition, children in the intervention and control groups received 2 regular 50-minute physical education sessions per week, as it is compulsory by law in Spanish schools.

**Conclusion:**

This study will determine the effect of an after-school physical activity intervention (MOVI-daFIT!), designed as a HIIT program, on reducing fat mass and cardiovascular risk, and improving fitness and cognition, including executive function and academic achievement.

## 1 Introduction

During the last decades, children's sedentary behaviors have been continuously increasing.^[1,2]^ As the World Health Organization (WHO) reports, 81% of adolescents do not meet the recommended 60 minutes per day of moderate-to-vigorous intensity physical activity.^[3]^ Children have reduced the time spent on physical activity within the school setting, where academic subjects other than physical education have been progressively gaining importance^[4]^; furthermore, screen-based activities have replaced most time spent for leisure physical activity.^[2]^ In contrast, growing scientific evidence recommends increasing physical activity time to achieve benefits on children's health by counteracting the consequences of sedentary behaviors.^[5]^

Physical inactivity is one of the leading factors contributing to the worldwide increase of overweight/obesity rates.^[6]^ In addition, the increase of children's physical inactivity rates is coupled with a decrease in their cardiorespiratory fitness levels.^[7]^ Both overweight/obesity and low levels of fitness are associated with not only children's physical health disorders such as hypercholesterolemia, hypertension, hyperinsulinemia, and worsening subclinical arteriosclerosis markers,^[8–13]^ but also low scoring on cognition, academic achievement, self-esteem, and health-related quality of life.^[14,15]^

Because children spend a lot of time in school, this has been proposed as a good setting to promote healthy habits that decline the negative consequences of physical inactivity.^[16]^ The school environment offers a permanent platform to access people at early ages, a key period of biological and social life in which physical activity interventions could produce positive short- and long-term benefits, and behavioral changes that tend to persist from infancy through adolescence to adulthood.^[5]^ School-based physical activity interventions have demonstrated positive effects to reduce body fat and cardiovascular risk factors.^[17]^ Furthermore, benefits of physical activity programs on children's cognition, metacognition, and academic performance have been reported.^[18,19]^

Most of the previous interventions were aimed at achieving international recommendations for physical activity. With this aim, in recent years high-intensity interval training (HIIT) programs have been implemented, which have demonstrated to be time-efficient and to easily fit into children's patterns of spontaneous exercise.^[20]^ HIIT programs have reported greater positive effects than other physical activity programs on cardiorespiratory fitness and cardiometabolic parameters, and are particularly effective in lowering blood pressure and cardiovascular disease biomarkers.^[20,21]^ However, their impact on other health parameters, such as body mass and fat, and cognition, remains unclear.

## 2 Aims

The purpose of this paper is to describe the study design and protocol for a HIIT physical activity school-based program named MOVI-daFIT!. The objective of this study was to assess the effectiveness of the MOVI-daFIT! intervention in children from 9 to 11 years old on improving physical fitness, and reducing fat mass and cardiovascular risk; and improving executive function and academic achievement.

## 3 Methods

### 3.1 Design

This study was designed as a cluster randomized controlled trial with 2 parallel groups. The CONSORT 2010 statement: extension for cluster trials^[22]^ will be used to conduct and report results of this trial.

### 3.2 Recruitment and allocation

**Figure 1:**
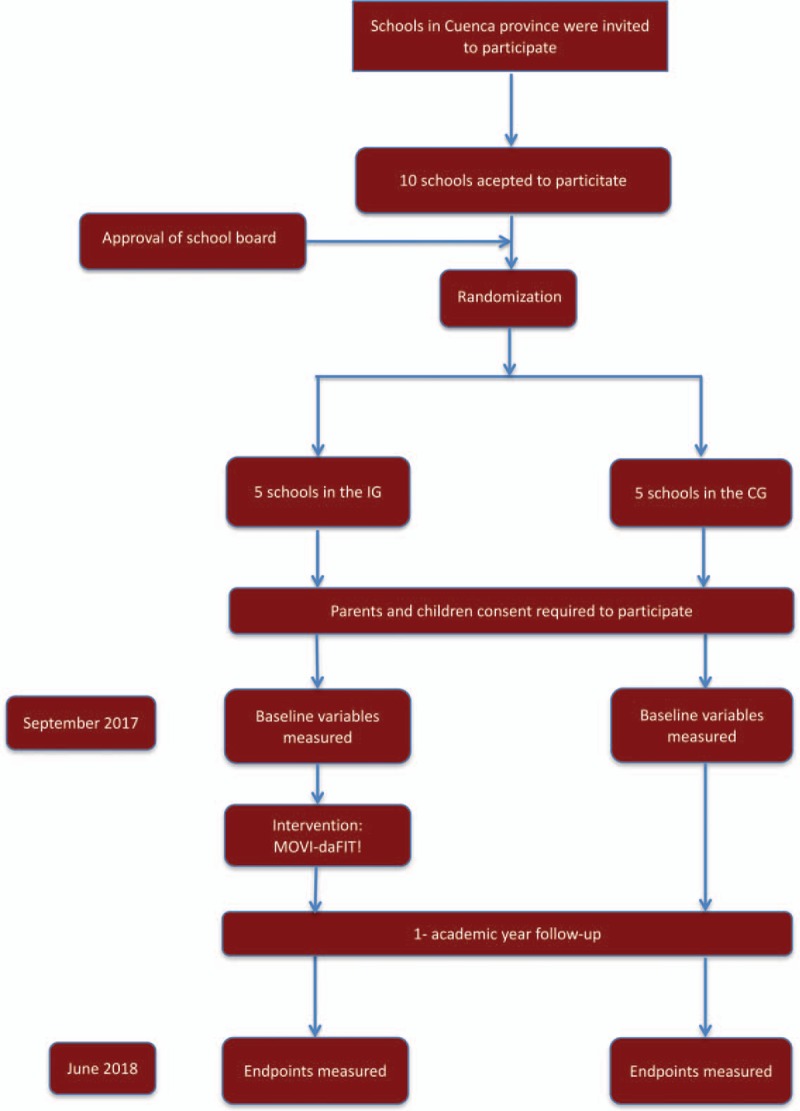
Flow chart of trial participants. CG = control group; IG = intervention group.

The Clinical Research Ethics Committee of the ‘Virgen de la Luz’ Hospital in Cuenca approved the study protocol. The development of this project was supported by the Department of Education and Science of the Junta de Communities of Castilla-La Mancha (Spain), which sent a letter informing each school that agreed to participate about the study. After that MOVI-daFIT! researchers provided information about the objectives and methods of the study to the head teacher, the school board, and the physical education teachers of the schools. The consent of the School Council, board of community participating in school management, was required to participate in MOVI-daFIT!. Finally, 10 schools from 10 towns in the province in Cuenca, Spain, agreed to participate (Fig. 1).

After approval from School Councils, the schools were randomly assigned to either the intervention group (IG) or the control group (CG), by using the statistical package StatsDirect.

### 3.3 Participants and study sites

In all schools, all children belonging to the fourth and fifth grades of primary school (9–11 years old) were invited to participate. Parents were invited to a meeting in which researchers provided complete information about the objectives and procedures of the study. Signed informed consent from parents was compulsory for the children whose parents decided that they will participate in MOVI-daFIT!. Parents were encouraged to take children's opinion into consideration for this decision.

Children's clinical parameters information was sent to their parents after each measurement. In addition, appropriate recommendations were suggested when abnormal values were detected. Finally, parents had access to the MOVI website (www.movidavida.org), where they could post any questions to the MOVI group and share videos or photos.

### 3.4 Inclusion and exclusion criteria

To participate in the study, schools were required to have at least one full classroom of fourth grade and another of fifth grade. Children were excluded when presenting Spanish learning difficulties; teachers or parents reported any child's serious physical or mental disorders which could impede participation in the activities of the program; or pediatricians reported any child's chronic disorder such as heart disease, diabetes, or asthma that could prevent participation in the activities of the program.

### 3.5 Intervention

#### 3.5.1 MOVI-daFIT!: description

This program includes recreational and noncompetitive physical activities, based on traditional games, but using a HIIT protocol adapted to children's age. A detailed description of the playground games used in the exercise sessions is freely accessible at www.movidavida.org. The same games as the MOVI study were used, but several adaptations to increase physical intensity were made. For example, in a game of chase when a child is caught, he/she cannot stand still waiting for another child to save him/her but must do jumping jacks, running on the spot, squats, and so on. An example session can be found in Table 1.

**Table 1:**
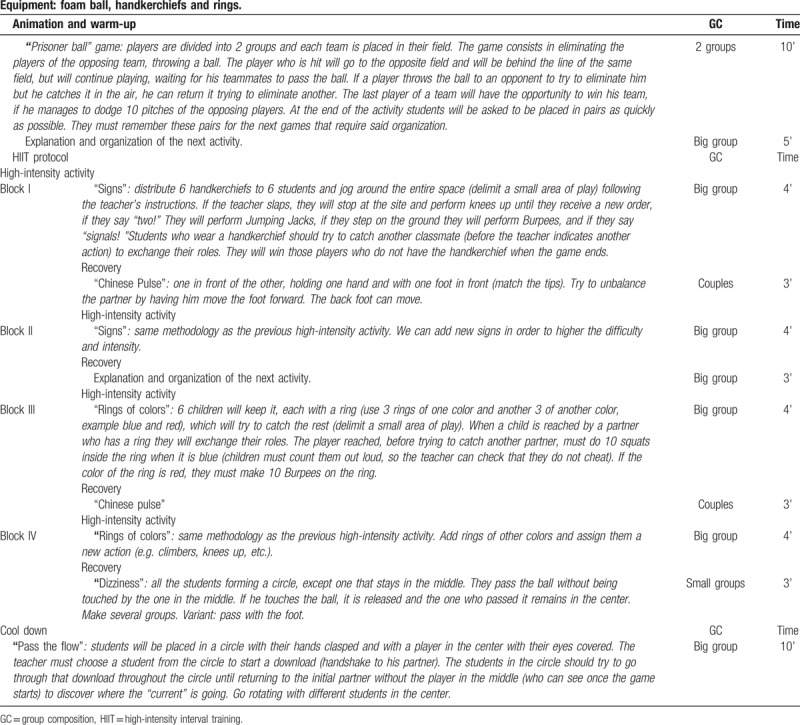
MOVI-daFIT! session type.

The program was implemented from October 2017 to May 2018, including a total of 102 sessions in each school. For statistical analyses, children who attended ≥80% of the MOVI-daFIT! sessions were considered to have received the intervention.

#### 3.5.2 MOVI-daFIT!: prescribed amount/intensity

**Figure 2:**
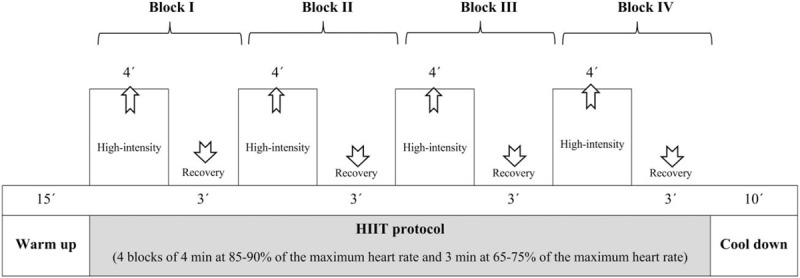
Session design MOVI-daFIT!.

Children were involved in 60-minute after-school sessions 4 times a week developed within the school setting.^[23]^ Each session consisted of 15 minutes of set-up and warm-up games, followed by 28 minutes of games using the HIIT protocol, in which a 4-minute game of high-intensity activity (at 85%–90% of the maximum heart rate, approximately 178–190 ppm) was followed by a game of recovery activity lasting 3 minutes (at 65%–75% of the maximum heart rate, approximately 136–147 ppm), and this sequence was repeated 4 times. Finally, children played a 10-minute low-intensity game for cool down (Fig. 2). Sometimes, the games were repeated to minimize the time spent explaining. To ensure that children reached the intensity of the planned physical activity, children's heart rates were randomly monitored each session. When the planned intensity per session was not reached, trainers modified the exercise characteristics to achieve the daily target (increasing the number of children catching, reducing the playing space or including new rules). On the contrary, if the children exceeded the desired intensity, there was no recovery game, and children rested standing while listening to the explanations for the next game.

To increase the workload, after the 12th week of the program, the session's protocol increased the HIIT time to 32 minutes, which included 4 blocks of alternating 5 minutes of high-intensity activity with 3 minutes of recovery activity. During this phase, the warm-up and the cool down lasted approximately 10 minutes each. This volume and intensity were maintained until the end of the program.

#### 3.5.3 MOVI-daFIT!: design and teacher training

The MOVI-daFIT! program was designed by 2 physical activity and sport sciences graduates, and implemented by trainers with technical qualifications in physical activity and sports who were blinded to the participants group assignment. Trainers learned the MOVI-daFIT! program during a 1-day training session to standardize the implementation of the program. The workshop was conducted by the main researcher of the project and the researchers responsible for the design of the physical activity program. The main objective was to engage, inspire, and equip trainers with the necessary skills to perform the MOVI-daFIT! protocol.

#### 3.5.4 MOVI-daFIT!: monitoring and adherence

To improve adherence to the program, participants attending ≥80% of the sessions received small gifts depicting the logo of the program's mascot as a reward (t-shirts, fidget spinners, etc.). A telephone number and an email address were available for parents and teachers to keep them informed about the development of the program. Monthly contacts with monitors were held by phone and e-mail to obtain information on the attendance of children to the program. Moreover, a visit to the schools was made to assess program performance and conduct satisfaction surveys with children. Finally, the monitors were responsible for accounting adverse effects derived from the program and recording the reasons for dropout.

Children in both the IG and CG received, as it is compulsory by law in Spanish schools, 2 regular 50-minute physical education sessions per week.

## 4 Outcome measures

Baseline and postintervention measurements corresponded with the beginning and the end of the school year (September 2017 and June 2018, respectively). Table 2 summarizes the variables measured.

**Table 2:**
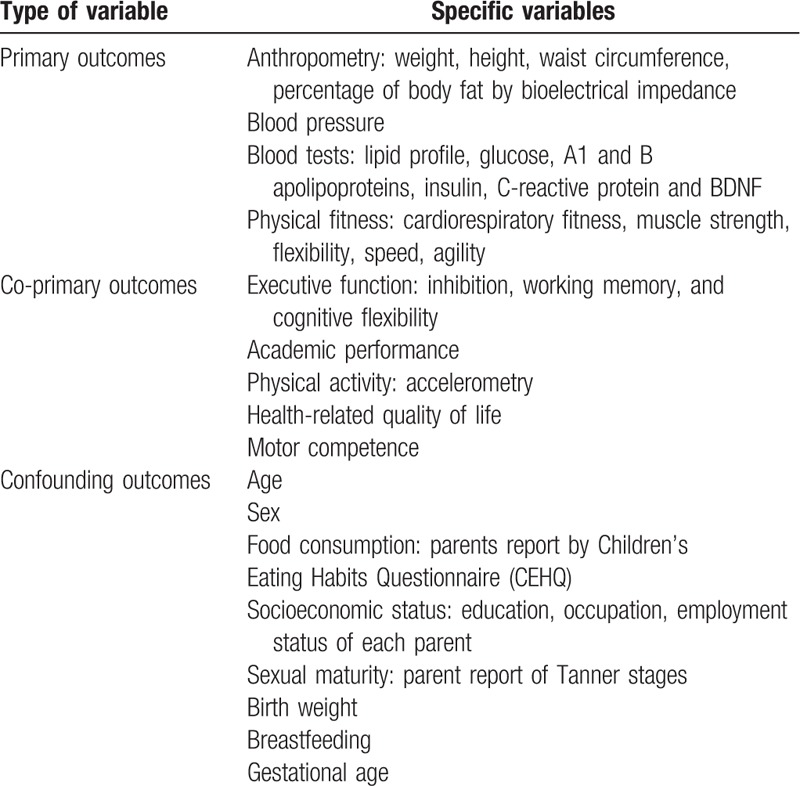
Study variables.

The measurements were made at school by evaluators who were previously trained to standardize the measurements and blinded to the group in which participants were allocated.

### 4.1 Primary outcome

*Anthropometric variables* were measured twice, and their average was considered for the analyses. Weight was measured using a scale (Seca 861) with the child in light clothing and barefoot. For height, a wall stadiometer (Seca 222) was used with children barefoot and upright while the sagittal midline touched the backboard. Children had to look straight ahead, and the line of sight had to be parallel to the floor.

*Body composition.* Body mass index (BMI) was calculated as weight (kg)/height (m)^2^. Waist circumference was considered as the mean of 3 measurements using a flexible tape at the midpoint between the last rib and the iliac crest at the end of a normal expiration. The percentage of body fat and fat-free mass were measured by using an 8-electrode Tanita Segmental-418 bioimpedance analysis system (Tanita Corp., Tokyo, Japan).^[24]^ Body fat and fat-free determinations were performed under controlled temperature and humidity conditions. In addition, the measurements were done before breakfast, after urination, and after a 15-minute resting period.

*Blood pressure.* Systolic and diastolic blood pressure and heart rate were measured in a quiet place and after a 5-minute resting period using an OMRON-M5-I device (Omron Healthcare UK Ltd.),^[25]^ and cuff size was used according to the child's arm circumference. We considered for analyses the mean of the 2 measures separated by 5 minutes each.

*Biochemical determinations.* Blood samples were drawn from the cubital vein between 9.00 and 10.00 am, and after a 12-hour fasting period. Two aliquots from each sample were frozen for future determinations that could be of interest for parents.

*Lipid profile.* Cholesterol, triglycerides, high-density lipoprotein cholesterol (cHDL) and low-density lipoprotein cholesterol (cLDL), glucose, apolipoproteins A1 and A2, insulin, glycated hemoglobin A1c (HbA1c), C-reactive protein, and brain-derived neurotrophic factor (BDNF) were determined. FPG and lipid profile measurements were performed using the Cobas 8000 Roche Diagnostics system. Insulin was determined with the Architect i2000 Abbot Diagnostic system. C-reactive protein was measured using the Cobas 6000 Roche Diagnostic system and HbA1c by high-performance liquid chromatography (HPLC) with the analyzer ADAMS HA 8180 V Menarini Diagnostic, which was standardized for the Diabetes Control and Complications Trial (DCCT), and the International Federation of Clinical Chemistry and Laboratory Medicine (IFCC). Samples were refrigerated when they were drawn farther than 75 minutes away from the laboratory. Finally, biochemical determinations allowed for estimating insulin resistance using the HOMA model and determining the metabolic syndrome index.^[26]^

*Physical fitness parameters* were measured after a 4-minute warm-up, using the PREFIT battery^[27,28]^ as follows:

– *Cardiorespiratory fitness* was measured using the Course Navette test (20-m shuttle run test). Two lines 20-m apart were painted on the floor. Children were encouraged to run back and forward following the sound signal of a prerecorded tape. This sound started with a cadence of 6.5 km/h and increased in 0.5 km/h each minute. Children failed the test when they did not reach the lines before the signal sounded twice. Maximal oxygen intake was calculated by applying the preschool-adapted 20-m shuttle-run (PREFIT) formula.^[29]^
– *Muscle strength* was assessed based on upper body and lower explosive body strength.

A digital dynamometer with adjustable grip TKK 5401 Grip-DW (Takeya, Tokyo, Japan) was used to measure upper body strength in kilogram. The test was performed twice with the right hand and twice with the left hand; the mean average of the 4 measurements was calculated.

The standing long jump was used to measure lower explosive body strength. Children stood behind a line with their feet approximately shoulder width apart and jumped as far as possible with both feet. The test measures the distance in centimeters from the starting line to the back of the children's heels. The best of 3 trials was recorded.

– *Speed ability* was assessed using the speed–agility test 4 × 10 shuttle run test. Children ran as fast as they could 4 times between 2 lines 10-m apart. The best of 2 trials in a 5-minute interval was recorded in seconds. For analyses, this variable was multiplied by −1, as less time represents better results.
– *Flexibility* was measured by the sit-and-reach test. Children sat on the floor with their legs out straight ahead and feet placed flat against a box. The maximum distance the children could reach with their fingertips by flexing the trunk keeping the fingers level and without flexing the knees was measured. The best mark from 3 attempts was used for analyses.^[30]^

### 4.2 Co-primary outcomes

#### 4.2.1 Executive functions

The 3 core executive functions ^[31]^ (i.e., inhibition, working memory, and cognitive flexibility) were measured by using the NIH tool box.^[32]^ All measurements were performed using the digital format test, and was administered to the children individually and in a quiet room.

In brief, inhibition was measured using an adapted version of the Eriksen Flanker Task.^[33]^ The version included a practice block of 4 trials that children passed if they got ≥3 trials. If so, a 20-trials block is presented consisting of a pseudorandom sequence of congruent and incongruent trials. Validation procedures previously reported were used to calculate accuracy percentage, and reaction time on congruent and incongruent trials; in addition, raw scores were obtained.^[34]^

Working memory was measured using the List Sorting Working Memory Test.^[34]^ Children were presented a series of illustrated pictures, after that they were asked to verbally repeat the names of the pictures in order of size, from smallest to largest. The number of objects in the series increased by one each time. Two list versions were presented, the first presenting items in one category (i.e., animals), and the second presenting items in 2 categories (i.e., animals and food). In the second version, children were asked to remember and organize items by category and size.

Cognitive flexibility was measured using the Dimensional Change Card Sort test.^[33]^ This tool presented a stimulus by “color” or “shape,” and children were asked to adapt their response according to the relevant dimension. First, a 4-trial practice block was presented, from which children needed to pass 3 to get the mixed 30-trial block. During the mixed block, “color” and “shape” trials were presented in a pseudorandom order. Validation procedures previously reported were used to calculate accuracy percentage and reaction time on pre-switch and post-switch trials; in addition, raw scores were obtained.^[33]^

*Academic achievement* was assessed through school records on language and mathematics academic subjects. Scores at the end of the 2016 to 2017 and the 2017 to 2018 school years were considered as baseline and final measurements, respectively.

*Physical activity.* In a subsample of 500 randomly selected children, physical activity was objectively measured using GENEActive accelerometers (ActivInsights) for 7 consecutive days (including nights), with a fixed frequency of 30.0 Hz to collect raw acceleration data measured in “g” for each movement axis (*x*, *y*, and *z*), to measure the physical activity of children in the IG and the CG. Data were stored directly in the memory of the device and expressed in units of milligram (1000 mg = 1 g = 9.81 m/s^2^).^[35]^ We considered as valid measurements those of ≥5 days, including 1 weekend day.

*Health-related quality of life* was measured using the validated Castilian version of KIDSCREEN-27.^[36]^ Children and parents were asked to separately complete this questionnaire to compare results.

*Motor competences* were assessed using 5 tests that evaluate gross motor skills (2 for ‘aiming and catching’ and 3 for ‘static and dynamic balance’) of the Movement Assessment Battery for Children for children from 7 to 10 years old. ^[37]^

### 4.3 Confounding variables

The following were considered as confounding variables:

- Food consumption was estimated using the Children's Eating Habits Questionnaire (CEHQ)^[38]^ completed by parents.
- Family socioeconomic status was estimated using the Spanish Epidemiology Society Scale. Mothers and fathers reported their respective educational levels and employment status, and an index was calculated considering both.^[39]^
- Parents reported sexual maturation identifying their children's pubertal status on figures based on the Tanner stages.^[40,41]^
- Birth weight, breastfeeding, gestational age, and parents’ information (weight and height) were also collected.

## 5 Data analysis and management

### 5.1 Sample size

The sample size was estimated by considering a 0.3 difference mean change between the IG and the CG in cardiorespiratory fitness, as has been reported in previous studies.^[42]^ The estimates were calculated considering an alpha error of 0.05 and statistical power of 0.80. In addition, a mean of 40 children was considered in each classroom with a 15% dropout rate. For sample size calculation, optimal design software was used and Donner and Klar's models were considered.^[43]^

### 5.2 Analyses of outcomes

After measurements, participants’ data will be entered into a database by 2 independent researchers. Blinding on handling data will be assured by separating measurements values from general participant's information. Statistical analyses will be performed in 3 waves. First, the effectiveness of the randomization processes will be checked by exploring outlier values, and the normal distribution of the variables using the Kolmogorov Smirnov test and graphical procedures (normal distribution plots). After checking the truthfulness of outliers and extreme values, these will be winsorized using the 1st percentile and 99th percentile of the distribution of variables. Before conducting the analyses, and after considering the missing at random or missing completely at random nature, children with missing data will be imputed using chained equations.

Second, mixed regression models^[44]^ will be used to assess the change between the baseline and final variable measurements. For these analyses, each variable will be considered as an independent variable, and the intervention as a fixed effect. In addition, the analyses will be adjusted for baseline data, age, sex, and school (cluster).

Finally, the previous analyses will be done using propensity score methods for comparing the IG and the CG in the scenario in which IG subjects have the same characteristics as the CG ones at the baseline. This statistical method allows each child in the IG to be matched with a child in the CG, by using a variable as reference.

Results will be considered statistically significant at *P* < 0.05. The analyses will be performed using version 9.2 of STATA14. Finally, statisticians conducting the analyses will be blinded to the participant's allocation.

## 6 Discussion

This paper states the rationale and methods of a cluster randomized trial aimed at testing the effectiveness of an after-school physical activity intervention (MOVI-daFIT!), designed as a HIIT program, on reducing fat mass and cardiovascular risk, and improving fitness and cognition, including executive function and academic achievement.

Since sedentary time and overweight prevalence among children have been increasing during the last decades, developing physical activity programs aimed at reducing both are a political interest and public health necessity.^[45]^ For this purpose, schools are suitable places to implement programs based on behavioral changes,^[5,16]^ but additional evidence is needed on how to benefit from the effects of physical activity on children's physical and mental health within these settings.

Previous physical activity programs have demonstrated some positive effects on cardiovascular risk factors, for example, blood pressure, cholesterol, waist circumference, and body fat^[46]^; however, these changes do not seem to be translated into lower BMI or overweight/obesity prevalence.^[47]^ Furthermore, physical activity programs have reported positive effects on children's cognition, metacognition, and academic performance.^[18,19]^ The benefits of physical activity programs on children's physical and mental health seem to be promoted by improvements on children's fitness,^[9]^ but it should be highlighted that a specific description of the physical activity characteristics (i.e., frequency, intensity, and duration) and their combination associated with greater health improvements is still needed. In addition, there is a lack of evidence on how the deliberated manipulation of task complexity impacts children's cognition.^[18]^

Echoing these calls, we have developed the MOVI-daFIT! program to translate into practice the WHO's physical activity children's recommendations.^[23]^ Each session was carefully detailed in advance and designed to achieve the moderate-to-vigorous daily recommendations of physical activity. Children were monitored to ensure the daily-intensity goal and the activities programmed per session were modified based on children's reports. Furthermore, activities included cognitive and coordinative demands needed to foster the positive effects of physical activity on children's cognition and to achieve the desired intensity. This approach helped us design more realistic physical activity sessions that could better fit into children's physical activity patterns and enhance the usefulness and transferability of our results.

Some strength points of this program are that it is developed within the school setting using school facilities, a familiar environment for children, but without requiring changes in the curriculum; the physical activity program takes parents’ opinions and preferences into consideration without overloading them; furthermore, it includes programmed and structured activities, which are easily reproducible; and activities are designed so each child reaches the daily goal without promoting competitiveness.

We should recognize some limitations. Although this is a randomized controlled trial, children, parents, and teachers could not be blinded regarding the allocation group and some bias could be derived from this fact. We tried to reduce this bias through the cluster-randomized design. In addition, the measurements and intervention program sessions were standardized by training the staff, but some variability could not be neglected. Finally, a program based on physical activity was designed without considering diet interventions, although diet behavior information was collected. We followed previous research considering that energy intake is not the main cause of overweight prevalence and that diet interventions could increase the obese/overweight prevalence.^[48,49]^

## Acknowledgments

We thank all schools, families, and pupils for their enthusiastic participation in the study.

*Aditional information section*: Clinicaltrials.gov identifier (NCT number): NCT03236337.

*On behalf of*: Carlos Berlanga-Macías, Blanca Notario-Pacheco, Ana Díez-Fernández, María Jesús Pardo-Guijarro, Marta Nieto-López, Alberto González-García, Jorge Cañete García-Prieto, Ana Torres-Costoso, Antonio García-Hermoso, Caterina Pesce, and Ricardo Cuevas-Campos.

## Author contributions

VMV and MSL designed the study. VMV was the principal investigator and guarantor. MSL, CAB, ICR, and VMV were the main coordinators of the study. CAB, ICR, DPPC, MGM, JAMH, VMM, and EPM conducted the study. ICR and VMV gave statistical and epidemiological support. VMV wrote the article with the support of MSL and CAB. VMV obtained the funding, with the assistance of MSL. All authors established the methods and questionnaires, provided comments on the drafts, and have read and approved the final version.

**Conceptualization:** Vicente Martínez-Vizcaíno, Celia Álvarez-Bueno, Iván Cavero-Redondo, Mairena Sánchez-López.

**Data curation:** Celia Álvarez-Bueno, Miriam Garrido-Miguel, Jose Alberto Martínez-Hortelano.

**Formal analysis:** Iván Cavero-Redondo, Diana P Pozuelo-Carrascosa.

**Funding acquisition:** Vicente Martínez-Vizcaíno, Enrique Prada de Medio, Mairena Sánchez-López.

**Investigation:** Celia Álvarez-Bueno, Diana P Pozuelo-Carrascosa, Miriam Garrido-Miguel, Jose Alberto Martínez-Hortelano, Vanesa Martínez-Madrid, Enrique Prada de Medio, Mairena Sánchez-López.

**Methodology:** Vicente Martínez-Vizcaíno, Iván Cavero-Redondo, Miriam Garrido-Miguel, Jose Alberto Martínez-Hortelano, Mairena Sánchez-López.

**Project administration:** Celia Álvarez-Bueno, Diana P Pozuelo-Carrascosa.

**Supervision:** Vicente Martínez-Vizcaíno, Iván Cavero-Redondo, Diana P Pozuelo-Carrascosa, Miriam Garrido-Miguel, Jose Alberto Martínez-Hortelano, Vanesa Martínez-Madrid, Enrique Prada de Medio, Mairena Sánchez-López.

**Writing – original draft:** Vicente Martínez-Vizcaíno, Celia Álvarez-Bueno, Iván Cavero-Redondo, Jose Alberto Martínez-Hortelano, Vanesa Martínez-Madrid, Enrique Prada de Medio.

**Writing – review and editing:** Vicente Martínez-Vizcaíno, Celia Álvarez-Bueno, Iván Cavero-Redondo, Miriam Garrido-Miguel, Mairena Sánchez-López.
